# Transcription factor MEF2D regulates aberrant expression of ACSL3 and enhances sorafenib resistance by inhibiting ferroptosis in HCC

**DOI:** 10.3389/fphar.2024.1464852

**Published:** 2024-12-18

**Authors:** Xue Li, Shimin Chen, Yuanyuan Shi, Yuanjing Wang, Xuanzhe Wang, Qian Lin, Chao Wu, Wenshuo Fang, Peng Sun, Leina Ma

**Affiliations:** ^1^ School of Basic Medicine, Qingdao University, Qingdao, China; ^2^ Qingdao Cancer Institute, The Affiliated Hospital of Qingdao University, Qingdao University, Qingdao, China; ^3^ Biomedical center, Qingdao university, Qingdao, China; ^4^ Hepatobiliary Surgery Department 1, Qingdao Central Hospital, University of Health and Rehabilitation Sciences, Qingdao, China; ^5^ Department of Clinical Laboratory, Maternity and Child Care Hospital, Weifang, China; ^6^ Department of Hepatobiliary and Pancreatic Surgery, The Affiliated Hospital of Qingdao University, Qingdao, China

**Keywords:** ACSL3, MEF2D, HCC, ferroptosis, sorafenib resistance

## Abstract

**Background:**

Sorafenib is a first-line treatment for hepatocellular carcinoma (HCC); however, acquired resistance often results in a poor prognosis, indicating a need for more effective therapies. Sorafenib induces cell death through an iron-dependent mechanism known as ferroptosis, which is closely associated with the onset and progression of HCC.

**Methods:**

This study investigated the role of ACSL3 in sorafenib resistance and ferroptosis in HCC. The expression of ACSL3 was analyzed in HCC tissues and cell lines. Ferroptosis levels and cell viability were assessed in ACSL3-silenced HCC cells treated with sorafenib. The regulatory relationship between the transcription factor MEF2D and ACSL3 was evaluated using promoter binding assays and gene expression analysis.

**Results:**

ACSL3 was aberrantly expressed in HCC and promoted the progression of non-alcoholic fatty liver disease (NAFLD) to HCC. Elevated ACSL3 expression inhibited ferroptosis and enhanced resistance to sorafenib. The transcription factor MEF2D directly regulated the upregulation of ACSL3 expression. MEF2D bound to the promoter regions of ACSL3 to enhance its transcription and negatively regulate ferroptosis in HCC.

**Conclusion:**

This study demonstrated for the first time that MEF2D regulated ACSL3 expression and mediated sorafenib resistance by inhibiting ferroptosis in HCC, providing a potential therapeutic target for improving HCC outcomes.

## 1 Introduction

Hepatocellular carcinoma (HCC) is the most common type of primary liver cancer (Forner et al., 2018). Sorafenib, a multi-targeted tyrosine kinase inhibitor, is widely used in the treatment of HCC; however, acquired resistance to this drug results in a poor prognosis for patients (Ladd et al., 2024; Gao et al., 2021). While HCC is commonly associated with hepatitis viral infections and alcoholic cirrhosis (Huang et al., 2023), Non-alcoholic fatty liver disease (NAFLD) has emerged as a significant concern over the past few decades due to its high global incidence among chronic liver diseases (Foerster et al., 2022; Ahmed et al., 2019).

NAFLD is a multi-system disease driven by metabolic syndrome and closely related to obesity, diabetes, and hyperlipidemia (Younossi et al., 2018; Committee of Hepatology, Chinese Research Hospital Association, 2019). The progression of NAFLD comprises two stages: non-alcoholic fatty liver (NAFL), which is characterized by simple steatosis, and non-alcoholic steatohepatitis (NASH), accompanied by steatosis, lobular inflammation, and fibrosis (Velliou et al., 2023; He et al., 2023). With the progression of liver fibrosis, especially cirrhosis and liver decompensation, the risk of HCC in patients with NAFLD increases rapidly (Lin et al., 2024; Shah et al., 2023).

Ferroptosis is a new type of programmed cell death characterized by the iron-dependent accumulation of high levels of reactive oxygen species (ROS) and lipid peroxides (LPO) (Jiang et al., 2021; Pope and Dixon, 2023). This disruption of the cellular redox balance ultimately leads to cell death (Xie et al., 2023; Seibt et al., 2019).

As a member of the long-chain acyl-CoA synthetase (ACSL) family, ACSL3 plays a crucial role in lecithin synthesis, lipid droplet formation, and the regulation of intracellular lipid homeostasis (Coleman et al., 2002; Poppelreuther et al., 2012). In addition, ACSL3 catalyzes palmitic acid and other fatty acids to generate acyl-CoA (Kageyama et al., 2013; Luo et al., 2023), which binds to membrane phospholipids and protects cells from ferroptosis. ACSL3 can also activate exogenous monounsaturated fatty acids (MUFA), thereby reducing the sensitivity of plasma membrane lipids to oxidation and effectively inhibiting ferroptosis within a few hours (Yang et al., 2022; Magtanong et al., 2019).

Myocyte enhancer factor 2D (MEF2D) is a transcription factor initially identified in muscle cells. Numerous studies have demonstrated that it serves as a key regulator of cancer-related gene transcription (Atkins et al., 2016; Di Giorgio et al., 2018). In liver cancer, overexpression of MEF2D is associated with tumor cell proliferation, invasion and metastasis, and immunosuppression (Ma et al., 2014; Yan et al., 2019; Xiang et al., 2020). It is critical in the progression of HCC. Furthermore, MEF2D is implicated in the progression of liver diseases, including cirrhosis (Wang et al., 2004).

In this study, we determined that ACSL3 was upregulated in NAFLD and HCC. Moreover, it played a key role in the progression from NAFLD to HCC by promoting hepatocyte injury and inflammation. We then demonstrated that ACSL3 overexpression inhibited ferroptosis and enhanced resistance to sorafenib therapy in HCC. MEF2D was the main regulator of ACSL3 gene transcriptional reprogramming in HCC. The aberrant expression of MEF2D-regulated ACSL3 was related to the poor prognosis of HCC patients.

## 2 Materials and methods

### 2.1 Animals

Female 5-week-old age C57BL/6 mice, weighing 18–25 g, were purchased from SPF (Beijing) BIOTECHNOLOGY Co. Ltd. One group (N = 3) was fed a standard chow diet (soybean meal, fish meal, Brewer’s yeast, vegetable oil, Bran, corn, wheat, vitamins, pantothenic acid, minerals). A second group (N = 3) was fed a high fat diet (standard feed 70% + lard 20% + cholesterol 5% + yolk 5%). All mice were provided with normal water (Soret et al., 2020). All mice were housed in a 12 h light–12 h dark cycle in a standard facility. At 6 weeks, livers were harvested, washed in saline solution, and formalin-fixed. All animal experimental procedures followed the guidelines of the Ethical Committee of the Medical College of Qingdao University (Animal Ethics Approval Number: QDU-AEC-2022097).

### 2.2 Patients and specimens of NAFLD-related HCC

Clinical information and surgical specimens were taken patients with NAFLD-related HCC from the Department of Hepatobiliary Surgery of Qingdao Central (Oncology) Hospital, Medical College of Qingdao University (Qingdao, China). All patients were diagnosed and treated for the first time and were not exposed to any prior treatment. The human sample collection procedure was approved by the Ethics Committee of the Medical College of Qingdao University. The informed consent was obtained before the collection of human specimens (Human Ethics Approval Number: QDU-HEC-2022055).

### 2.3 Drugs and reagents

Protease Inhibitor cocktail (Targetmol, cat#C0001), Phosphatase Inhibitor cocktail (Targetmol, cat#C0002), Phosphatase Inhibitor Cocktail II (Targetmol, cat#C0003), Bradford Assay Reagent (Thermo, Prod#1863028), Transfer Membrane (Merck Millipore Ltd., LOT:IPVH07850), Tris (Solarbio, Cat#T8060), Ammonium persulfate (Sinopharm Chemical Reagent Co. Ltd.), 30% Acrylamide/Bissoln (MDBio, lnc, Lot#F1110419), 1,2 Bis (dimethylamino)ethane-TEMED (Solaibio, Cas#110-18-9), Sorafenib (Meilunbio, Cat# MB1666), Erastin (MCE, Cat No.:HY-15763), Fer-1 (MCE, Cat No.: HY-100579), EntransterTM-R4000 (Engreen Biosystem Cat#4000-3), TriZol (SparkJade, Cat#AC0101-B), cDNA synthesis Mix (TRANS, Cat# AE311-04), HE Staining Kit (BASO, Cat#BA4025), Rhamsan gum (Sinopharm, Cat#10004160), OPTI-MEM medium 1X (Gibco, Cat#31985), Polymeric anti-rabbit IgG-HRP kit (BOSTER, Cat#SV0002), Polymeric anti-mouse IgG-HRP kit (BOSTER, Cat#SV0001), Endogenous peroxidase blockers (BOS-TER, Cat# AR1108), EDTA Antigen repair solution (10X) (BOSTER, Cat#AR0023), Anti-body dilutions (BOSTER, Cat#AR1016), PBS Bleach (BOSTER, Cat#AR0030), DAB Color Development kit (Yellow) (BOSTER, Cat#AR1022), Mayor hematoxylin (BOSTER, Cat#AR0005), Neutral gum (BOSTER, Cat#AR0038), Protein Marker 10-180kd (ABclonal, Cat#RM19001). Formalin (Biosharp, Lot 70081800), ACSL3 antibody (Santa cruz, 1:100, Cat# sc-166374), ACSL4 Rabbit pAb (ABclonal, 1:1,000, Cat#A6826), MEF2D antibody (Santa cruz, 1:500, Cat# sc-271153), GPX4 Monoclonal antibody (proteintech, 1:1,000, Cat No.: 67763-1-Ig), Ferritin heavy chain Polyclonal antibody (proteintech, 1:1,000, Cat No.: 11682-1-AP), Vinculin Monoclonal antibody (proteintech, 1:10,000, Cat No.: 66305-1-Ig), Mouse monoclonal [JDC-10] Anti-Human IgG Fc (Abcam, Cat#ab99757).

### 2.4 The hematoxylin and eosin (H&E) staining

Staining with the standard H&E staining technique in paraffin-embedded sections was performed according to the commercially available methods by the Boster Biological Technology center. The samples were processed by dewaxing the sections, hematoxylin sustaining for 3 min, the hydrochloride alcohol solution for 2 s dying after washing by water, staining with eosin solution for 1 s, dehydration by absolute ethyl alcohol for 2 s after washing by water. All the cell morphologic changes in liver tissue were observed under the OLYMPUS BX51 microscope and the images were collected by a Leica pathology fish slide scanner.

### 2.5 Immunohistochemistry (IHC) staining

IHC staining was performed according to the commercially available methods by the Boster Biological Technology center. ACSL3 expression was investigated using unstained, formalin-fixed, paraffin-embedded tissue sections and was detected using anti-ACSL3 antibody. The tissues were incubated with primary antibodies overnight at 4°C and then with an HRP-conjugated secondary antibody at 37°C. Whole slide imaging magnification was performed using BX51 OLYMPUS microscope and APERIO VERSA 8 of Leica slide scanner. Images were inspected using TEKSQRAY Digital Pathology viewer software.

### 2.6 Cell culture

Huh7 and PLC/PRF/5 cells were purchased from national collection of authenticated cell cultures (Shanghai, China) and maintained in Dulbecco’s Modified Eagle Medium (DMEM) medium (Gibco, Cat#SH8122258) supplemented with 10% fetal bovine serum (FBS), 1% streptomycin-penicillin (Gibco, Cat#15140-122). All cells used in this work were maintained in culture media at 37°C in a humidified atmosphere with 5% CO_2_.

### 2.7 Western blotting

Total proteins were extracted with RIPA lysis buffer containing protease and phosphatase inhibitors. The protein concentration was determined using a Bradford reagent kit. Proteins were separated by SDS-PAGE and transferred to polyvinylidene fluoride membranes. Membranes were blocked with 5% skim milk for 1 h, and then incubated with primary antibody at 4°C overnight. Membranes were then washed with Tris-buffered saline containing Tween 20, and incubated with horseradish peroxidase-conjugated anti-mouse or anti-rabbit IgG secondary antibodies for 1 h at room temperature, and finally developed with enhanced chemiluminescence kit. Vinculin served as the loading control. ACSL3 antibody (Santa cruz, 1:100, Cat# sc-166374), ACSL4 Rabbit pAb (ABclonal, 1:1,000, Cat#A6826), MEF2D antibody (Santa cruz, 1:500, Cat# sc-271153), GPX4 Monoclonal antibody (proteintech, 1:1,000, Cat No.: 67763-1-Ig), Ferritin heavy chain Polyclonal antibody (FTH1) (proteintech, 1:1,000, Cat No.: 11682-1-AP), Vinculin Monoclonal antibody (proteintech, 1:10,000, Cat No.: 66305-1-Ig).

### 2.8 Small interfering RNA (siRNA) transfection

ACSL3 siRNA sequences included sense (5′-3′) CCU​GGA​UGU​GAU​ACU​UUA​GAU and antisense (5′-3′) AUC​UAA​AGU​AUC​ACA​UCC​AGG. These sequences were purchased from Sangon (Shanghai, China). Huh7 and PLC/PRF/5 cells were seeded in 6-well plates and maintained to 50% confluence prior to transfection siRNA and EntransterTM-R4000 were mixed in OPTI-MeM medium, and transfection complexes were placed at room temperature 15 min prior to adding to DMEM. After 6 h of transfection, media was exchanged and transfected cells were incubated for 48 h mRNA and protein expression levels of ACSL3 were evaluated using qPCR.

### 2.9 Construction of plasmids

Total RNA was extracted with Trizol reagent (Takara, Cat#9109) according to the manufacturer’s protocol. KOD -Plus- Neo (TOYOBO, Cat#KOD-401) was used to amplify the complete open reading frame (ORF) of ACSL3. Amplify ACSL3 gene was connected to pcDNA3.1 by Not I and Xba I restriction sites for expression in *E. coli*. In this study, Lv-GFP, Lv-MEF2D, Lv-scramble, and Lv-shMEF2D lentivirus construction details have been described in previous publications by our group (Ma et al., 2014).

### 2.10 Quantitative reverse-transcription polymerase chain reaction

Total RNA was extracted using the TriZol reagent and was measured using a Thermo Scientific NanoDrop instrument. Complementary DNA was synthesized using EasyScript^®^ One-Step gDNA Removal and cDNA Synthesis SuperMix. qRT-PCR was measured using Universal SYBR Green Fast qPCR Mix. All genes were normalized to GAPDH. mRNA expression levels of ACSL3, ACSL4, GPX4 and FTH1 were quantified using qPCR.

### 2.11 Cell viability detection

Viable cells were measured using a Cell Counting Kit-8 (CCK-8). In short, Specific treatment of cells were dispersed by Trypsin and seeded into 96-well plates at 4 × 103 cells/well. After 24 h of incubation, cells were treated with additional drugs (Sorafenib) at concentrations of 0 μM, 2 μM, 4 μM, 8 μM, 16 μM, 32 μM. After 24 h of treatment, the culture medium exchanged with 90 μL fresh complete medium and 10 μL CCK8 reagent (TargetMol, Boston, United States) were added to each well, followed by incubation for 1 h. The absorbance of each well was measured at 450 nm with a VICTOR^®^ Nivo™ microplate reader (PerkinElmer, Waltham, United States).

### 2.12 Detection of LPO and ROS levels

LPO levels were detected by Bodipy 581/591 C11 (MKBio, MX5211-1 MG, China). After the cells were pretreated with the drugs (sorafenib, erastin, fer-1), Bodipy 581/591 C11 was added and incubated for 20 min. Fluorescence was then measured at 595 and 510 nm using the SpectraMax i3x, Molecular Devices, United States. LPO levels were analyzed by calculating the ratio of 510/595 fluorescence intensity. The levels of ROS were detected using fluorescent probe DCFH-DA (Beyotime, S0033S, China). After the cells were pretreated with the drugs (sorafenib, erastin, fer-1), appropriate volume of diluted DCFH-DA was added and incubated for 20 min. Intracellular ROS can oxidize non-fluorescent DCFH to produce fluorescent DCF, and the level of intracellular ROS can be evaluated by detecting the fluorescence of DCF.

### 2.13 Chromatin immunoprecipitation (ChIP) assay

ChIP assay was performed using the ChIP assay kit (Beyotime, Cat No P2078). In brief, treated cells were cross-linked by incubation with 1% formaldehyde at 37° for 10 min and sonicated to break the DNA to a length ranging from 200 to 1,000 base pairs. Cell lysates were pre-clarified with protein A/G beads and then incubated overnight at 4°C with protein A/G beads coated with anti-MEF2D antibody (2 μg, Santa cruz). Anti-rabbit immunoglobulin G (IgG) was also used as a negative control. After extensive washing, the bead-bound immunocomplexes were eluted using elution buffer. To remove protein and genomic DNA cross-links, 5 M NaCl samples were added and heated at 65°C for 4 h, followed by proteinase K treatment and further incubation at 45°C for 1 h. The bound DNA fragments were subsequently purified and subjected to real-time PCR using specific primers.

### 2.14 Bioinformatics analysis

GSEA datasets GSE63067, GSE185051 and GSE180882 were used to investigate several KEGG metabolic pathways (https://www.ncbi.nlm.nih.gov/geo/). Expression profiles of ACSL3 in the murine livers were generated using the Tabula Muris database (https://tabula-muris.ds.czbiohub.org). The mRNA and protein expression of ACSL3 in liver cancer compared to normal tissues in the UALCAN databases (http://ualcan.path.uab.edu). The GEPIA2 database (http://gepia2.cancer-pku.cn/) was used to study the correlation between ACSL3 and hub genes expression. The prognostic value of ACSL3 and related genes was tested using the Kaplan-Meier Plotter (https://kmplot.com/analysis/). Gene association study and enrichment pathways were obtained using the Linked Omics database (http://linkedomics.org/). Transcription factor binding sites in the promoter regions of ACSL3 under study were carried out using AliBaba2.1 software (http://generegulation.com/pub/programs/alibaba2/index.html).

### 2.15 Statistical analysis

All data were analyzed using appropriate statistical analysis methods with GraphPad (Prism V.8.3.0) and data was presented as mean ± SEM.

## 3 Results

### 3.1 The high expression of ACSL3 promotes the progression of NAFLD to HCC and is associated with poor prognosis of HCC patients

**FIGURE 1:**
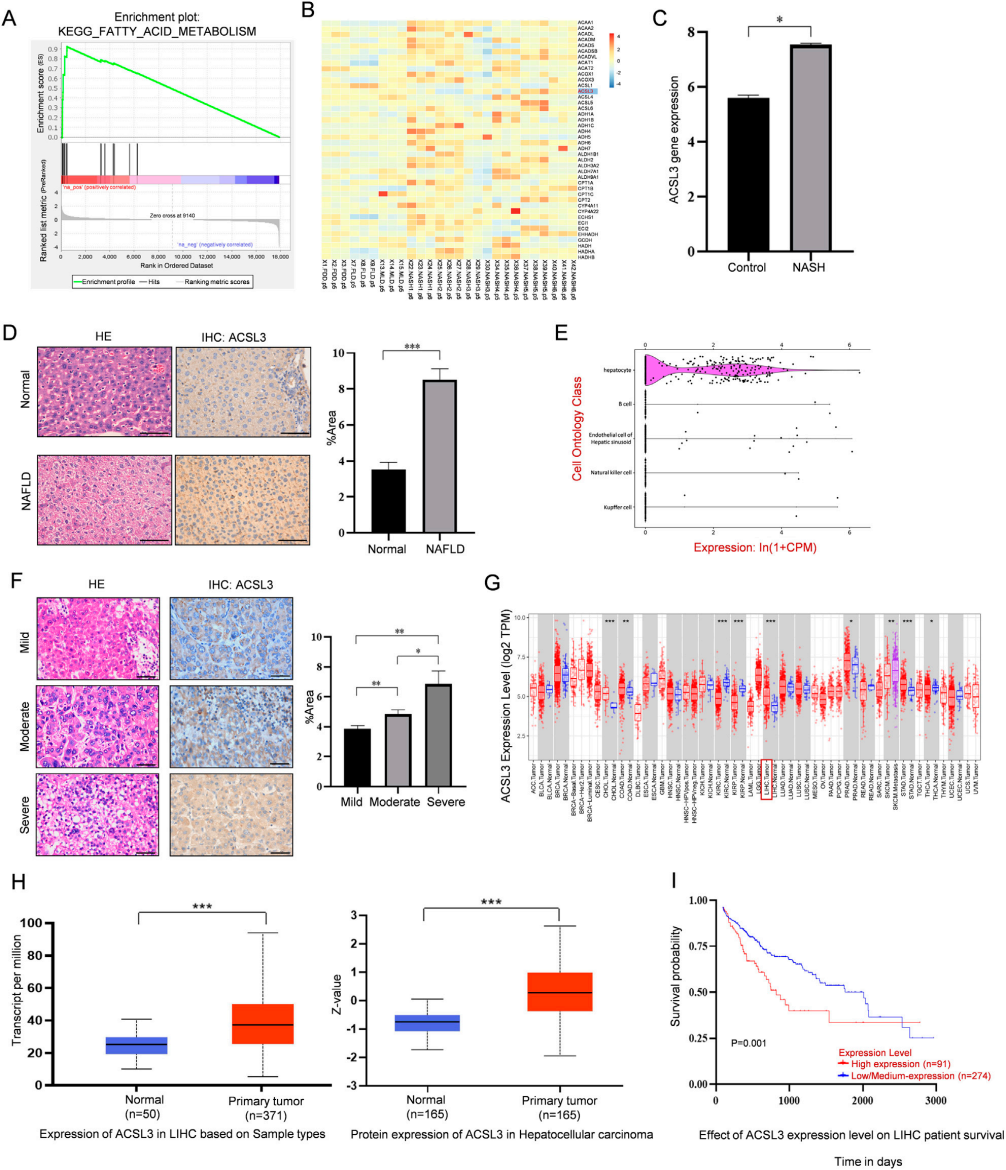
Abnormal expression and prognostic significance of ACSL3 in NAFLD and HCC **(A)** GSEA results showed the fatty acid metabolism pathways enriched in NAFLD tissues (GSE63067). **(B)** The heatmap revealed expression changes of the lipid metabolism-related ACSL3 gene in the expression profile of NAFLD (GSE185051). **(C)** The GEO data set showed changes in ACSL3 gene expression in NASH (GSE180882). **(D)** H&E and IHC staining revealed differences in liver injury and ACSL3 protein expression between normal liver tissues and NAFLD tissues. The number of positive cells in the IHC staining was quantitatively analyzed. Scale bar, 100 μm **(E)** Single-cell sequencing data of ACSL3 expression profiles in mouse tissues were sourced from the Tabula Muris database. **(F)** H&E and IHC staining revealed differences in liver injury and ACSL3 protein expression in human NAFLD-related (from mild to severe) HCC tissues. The number of positive cells in the IHC staining was quantitatively analyzed. Scale bar, 100 μm. **(G)** ACSL3 expression across multiple cancer types was analyzed using data from the TCGA database **(H)** The mRNA and protein expression levels of ACSL3 in liver cancer compared to normal tissues were analyzed using the UALCAN web portal **(I)** The prognostic significance of ACSL3 in HCC was analyzed using data from the TCGA database. ****p < 0.0001, ***p < 0.001, **p < 0.01, *p < 0.05.

Using Gene Set Enrichment Analysis (GSEA), we observed significant enrichment in fatty acid metabolism in tissues affected by NAFLD compared to healthy human liver tissues (Figure 1A). We also identified that ACSL3 was aberrantly expressed among genes linked to fatty acid metabolic pathways in NAFLD (Figure 1B), and ASCL3 mRNA levels were upregulated in NASH (Figure 1C). In NAFLD mice, H&E staining of liver tissue demonstrated an increase in scattered lipid vacuoles and compressed liver sinusoids compared to the control groups. Furthermore, IHC staining showed that increased ACSL3 protein expression (indicated by brown particles) in the liver tissue of NAFLD mice (Figure 1D). Single-cell sequencing of mouse tissues revealed that among the top five liver cell types, ACSL3 expression was predominantly confined to hepatocytes (Figure 1E).

To investigate the role of ACSL3 in the progression of NAFLD to HCC, we assessed cell morphology and ACSL3 antigenicity in human NAFLD-related HCC tissues, ranging from mild to severe. H&E and IHC staining showed that elevated levels of lipid vacuolization and overall ACSL3 expression in HCC tissues were positively correlated with the increased severity of NAFLD (Figure 1F). For information on patients with NAFLD-related HCC, we adopted the FLIP-SAF evaluation standard for the histological grading of NAFLD tissues (Supplementary Table S1).

By analyzing The Cancer Genome Atlas (TCGA), we found that the gene expression boxplot indicated significantly higher expression of ACSL3 in liver tumors compared to adjacent normal tissues (Figure 1G). Consistent with this, analysis using the UALCAN database also confirmed higher mRNA and protein expression of ACSL3 in liver cancer (Figure 1H). To assess the prognostic value of ACSL3 in HCC, we analyzed survival curves using TCGA database. Our analysis revealed that low expression levels of ACSL3 were associated with a higher survival rate in HCC patients (Figure 1I).

These results suggested that ACSL3 expression was elevated in NAFLD and HCC and promotes the progression from NAFLD to HCC, while high expression of ACSL3 was positively correlated with poor prognosis of HCC patients.

### 3.2 The aberrant expression of ACSL3 in HCC protects cells from ferroptosis

**FIGURE 2:**
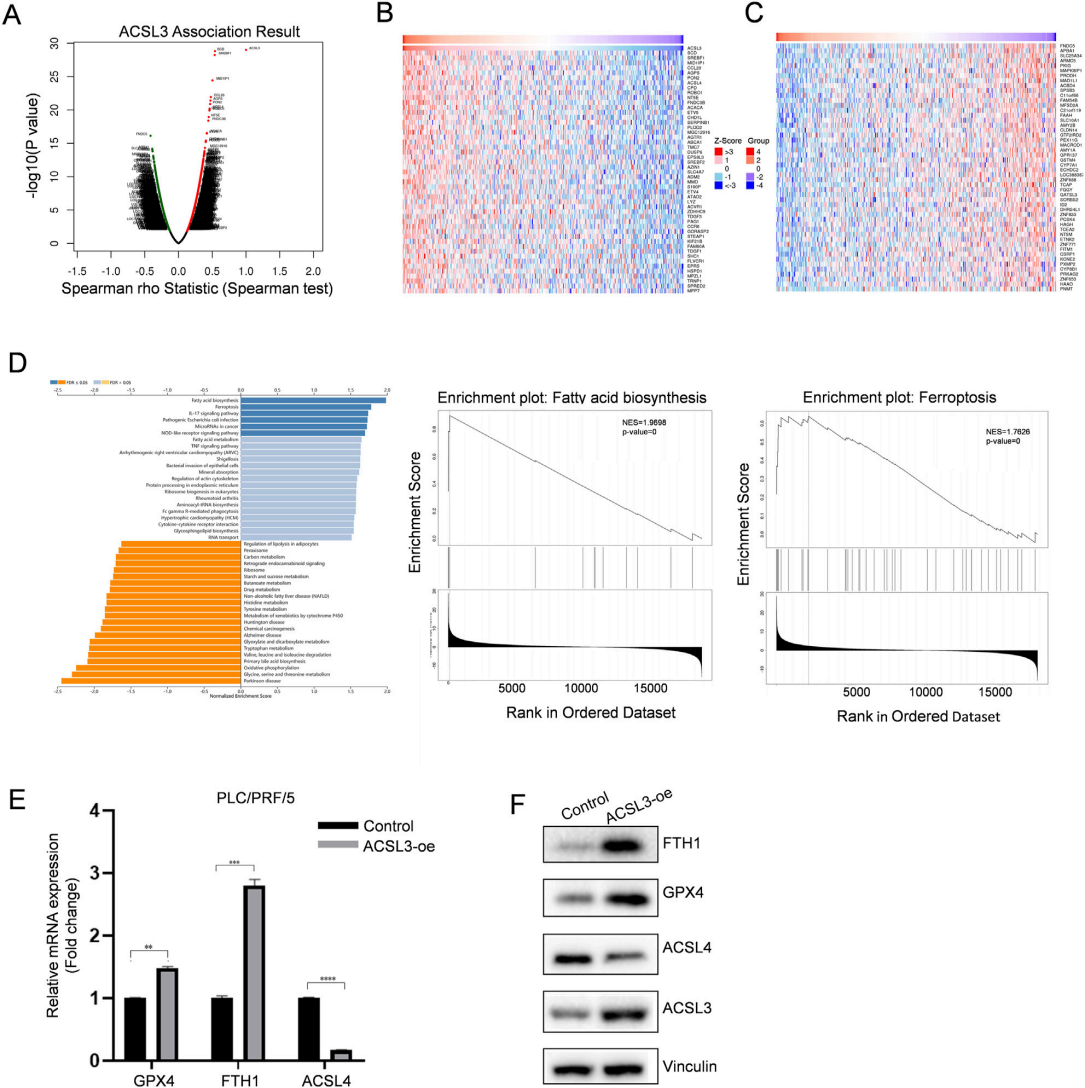
The aberrant expression of ACSL3 in HCC protects cells from ferroptosis. **(A)** Genes highly correlated with ACSL3 in HCC were analyzed using Pearson text **(B, C)** Heatmaps displayed the top 50 genes that are positively and negatively correlated with ACSL3 in HCC. **(D)** KEGG pathway analysis revealed the correlation between ACSL3 and fatty acid biosynthesis and ferroptosis signaling pathway **(E, F)** After transfecting ACSL3-oe and control plasmids into PLC/PRF/5 cells, the mRNA and protein expression levels of ACSL4, GPX4, and FTH1 were quantified using qPCR and Western blotting. Vinculin protein served as the internal control. ****p < 0.0001, ***p < 0.001, **p < 0.01, *p < 0.05.

To investigate the expression networks and signaling pathways related to ACSL3, we examined its co-expression profiles using the LinkedOmics database. As illustrated in the volcano plot (Figure 2A), red points indicated positive correlations with ACSL3, while green points represented negative correlations in HCC. Simultaneously, the heatmaps displayed the top 50 genes positively and negatively correlated with ACSL3 in HCC (Figures 2B, C; Supplementary Tables S2, S3).

GSEA indicated that the functional genes co-expressed with ACSL3 were primarily enriched in fatty acid biosynthesis and the ferroptosis signaling pathway (Figure 2D). Since ferroptosis is induced by the peroxidation of polyunsaturated fatty acids and regulation of lipid metabolism is a key determinant of ferroptosis sensitivity, the aberrant expression of ACSL3 in HCC may be closely linked to cellular ferroptosis. We evaluated the ferroptosis role of ACSL3 in HCC by overexpressing ACSL3 in PLC/PRF/5 cells, which have low endogenous levels of ACSL3. We then assessed the mRNA and protein levels of ferroptosis biomarkers by qPCR and Western blotting (Wu et al., 2019; Fang et al., 2021). Compared to the control group, the expression of the ferroptosis-positive regulator ACSL4 was downregulated, while the expression of the ferroptosis-inhibitory genes GPX4 and FTH1 was increased (Figures 2E, F).

These results collectively demonstrated that increased expression of ACSL3 played a significant role in ferroptosis within HCC.

### 3.3 ACSL3 enhances sorafenib resistance by inhibiting ferroptosis in HCC

**FIGURE 3:**
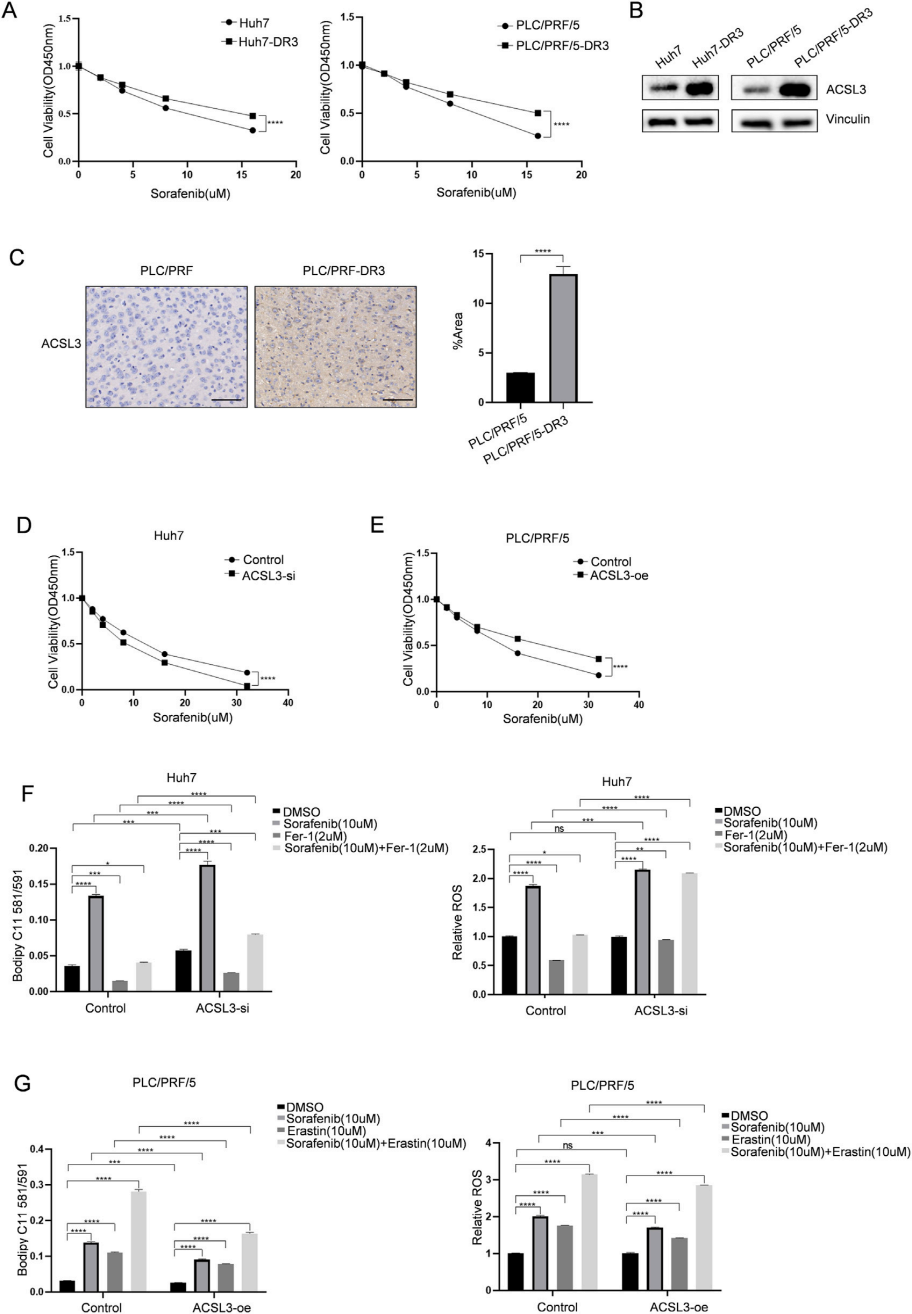
ACSL3 can inhibit ferroptosis and enhance sorafenib resistance in HCC **(A)** The cell viability of HCC cells and sorafenib-resistant HCC cells was assessed The cell viability of HCC cells and sorafenib-resistant HCC cells was assessed by CCK8 following treatment with a specific concentration of sorafenib. **(B)** Western blotting was used to detect and analyze ACSL3 protein expression in HCC and sorafenib-resistant HCC cells. Vinculin protein was used as an internal control **(C)** PLC/PRF/5 or PLC/PRF/5-DR3 cells were injected into male nude mice, and the tumors were treated with 50 mg/kg of sorafenib at regular intervals. IHC staining was used to detect ACSL3 expression in tumor tissues. The number of positive cells was quantitatively analyzed. Scale bar, 100 μm. **(D)** Following transfection of Huh7 cells with ACSL3-si or control plasmids, cell viability was evaluated using the CCK-8 assay after treatment with sorafenib **(E)** Following transfection of PLC/PRF/5 with ACSL3-oe plasmid or the control plasmids, cell viability was evaluated using the CCK-8 assay after treatment with sorafenib. **(F)** Following siRNA transfection, Huh7 cells were treated with sorafenib (10 µM) and fer-1 (2 µM) for 24 h. LPO levels were quantified using Bodipy 581/591 C11, and ROS levels were assessed using the DCFH-DA reagent. **(G)** Following transfection with overexpression plasmids, PLC/PRF/5 cells were treated with sorafenib (10 µM) and erastin (10 µM) for 24 h. LPO and ROS levels were measured using Bodipy 581/591 C11 and DCFH-DA, respectively. ****p < 0.0001, ***p < 0.001, **p < 0.01, *p < 0.05.

To determine the association of ACSL3 with sorafenib resistance in HCC, we established resistant cell lines, Huh7 (Huh7-DR3) and PLC/PRF/5 (PLC/PRF/5-DR3), by culturing them with increasing doses of sorafenib (Figure 3A). Western blotting showed that ACSL3 expression was increased in sorafenib-resistant HCC cells compared to the control sorafenib-sensitive cells (Figure 3B). Subsequently, PLC/PRF/5 and PLC/PRF/5-DR3 cells were injected subcutaneously into mice. After tumor formation, the mice were treated with sorafenib. IHC staining revealed that ACSL3 expression was significantly higher in tumors derived from drug-resistant cells compared to the control group (Figure 3C).

Subsequently, we transiently knocked down ACSL3 using specific siRNAs. As shown in Figure 3D, the activity of ACSL3-silenced Huh7 cells decreased following sorafenib treatment. By examining the levels of redox species, we validated the role of ACSL3 in ferroptosis and found that ACSL3 silencing consistently enhanced sorafenib-induced production of LPO and ROS. Additionally, we added the ferroptosis inhibitor fer-1 as a control during treatment (Figure 3F). Moreover, overexpression of ACSL3 in PLC/PRF/5 cells conferred resistance to sorafenib treatment and enhanced cell activity compared to the control group (Figure 3E). Cells with high ACSL3 expression were resistant to sorafenib-induced increases in LPO and ROS levels. The ferroptosis inducer erastin was also included as a control in the treatment (Figure 3G).

The results consistently demonstrated that the upregulation of ACSL3 expression in HCC protects against sorafenib-induced ferroptosis.

### 3.4 The transcription factor MEF2D binds to the promoter of ACSL3 and promotes ACSL3 expression

To understand the specific reasons of abnormal expression of ACSL3 in HCC, we conducted further exploration. Previous studies in our group have shown that MEF2D is overexpressed in HCC and sorafenib resistant-HCC. Abnormal expression of MEF2D enhances resistance to sorafenib therapy and is associated with poor prognosis in patients (Ma et al., 2021). Sorafenib is not only a multi-targeted tyrosine kinase inhibitor but also an inducer of ferroptosis, triggering oxidative stress in cells. We investigated whether there is a critical link between MEF2D, ACSL3 and ferroptosis in HCC.

**FIGURE 4:**
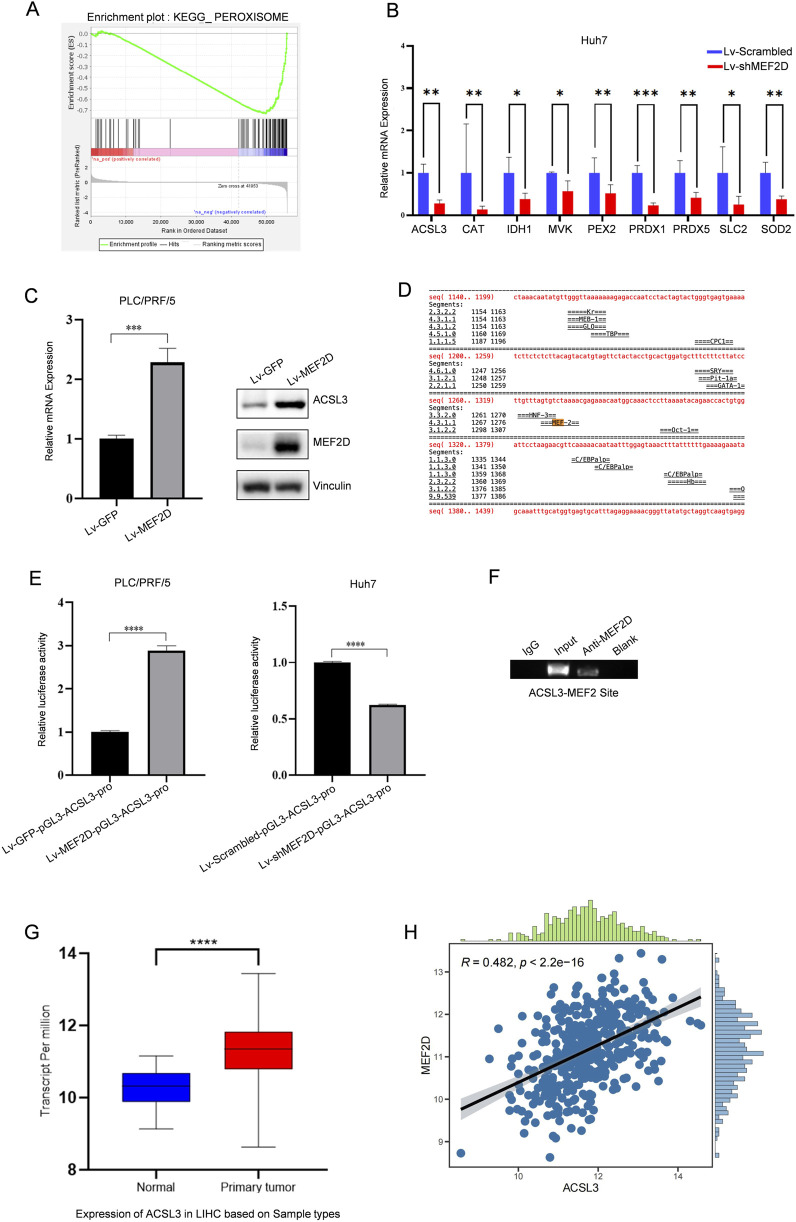
The transcription factor MEF2D regulates aberrant expression of ACSL3 **(A)** GSEA revealed enrichment of the peroxisome pathway in MEF2D-silenced HCC cells. **(B)** mRNA expression levels of ACSL3 and other oxidative stress-related candidate genes were analyzed in lv-shMEF2D-infected HCC cells and compared with controls **(C)** In PLC/PRF/5 cells infected with Lv-MEF2D or Lv-GFP, the mRNA expression of ACSL3 was quantitatively detected by qPCR, and the protein expression level was detected by Western blotting. Vinculin protein was used as an internal control. **(D)** Bioinformatic analysis for putative MEF2D-binding sites in the regulatory regions of ACSL3 was shown **(E)** Luciferase expression driven by ACSL3 promoter regions was measured in PLC/PRF/5 and Huh7 cells infected with Lv-MEF2D or Lv-shMEF2D. The folding changes of luciferasee relative activity in Lv-MEF2D- and Lv-shMEF2D infected cells were normalized to Lv-GFP- and Lv-scramble- infected cells, respectively. The data represent the mean ± SDs of three independent experiments. **(F)** A ChIP assay was performed to detect the binding of MEF2D to the potential MRE identified in the promoter regions of ACSL3. The IgG-incubated and blank groups were considered as negative controls, whereas the input fraction was the positive control **(G)** The mRNA expression levels of MEF2D in HCC tissues and normal tissues were analyzed using data from the TCGA database **(H)** The correlation between ACSL3 and MEF2D expression in HCC was analyzed using data from the TCGA database. ****p < 0.0001, ***p < 0.001, **p < 0.01, *p < 0.05.

Previously, our research group analyzed the gene differential expression in PLC/PRF/5 cells with or without MEF2D through RNA sequencing (Ma et al., 2014). GSEA revealed enrichment of the peroxisome pathway in MEF2D-silenced cells (Figure 4A). Peroxisomes are key mediators of cellular oxidative stress responses. Based on this, we found downregulated expression of several candidate genes related to oxidative stress in MEF2D-silenced HCC cells by qPCR. Notably, there was a significant difference in the expression of ACSL3, a gene associated with ferroptosis (Figure 4B). Additionally, the results indicated that in PLC/PRF/5 cells infected with Lv-GFP or Lv-MEF2D, the mRNA and protein expression levels of ACSL3 were positively correlated with MEF2D expression (Figure 4C).

To investigate the mechanism by which MEF2D directly regulates ACSL3, we used a bioinformatics approach to analyze the upstream regions of the ACSL3 transcription sites and identified a potential MEF2 recognition element (MRE) (Figure 4D). Subsequently, we performed a luciferase reporter assay in which luciferase expression was driven by the promoter of ACSL3. Overexpression of MEF2D enhanced the promoter activity of ACSL3. Conversely, knockdown of endogenous MEF2D levels also reduced luciferase activity driven by the ACSL3 promoter (Figure 4E). To further confirm the binding of MEF2D to the putative MRE in the upstream regions of the ACSL3 promoter, we conducted a ChIP assay in Huh7 cells. The results showed that the anti-MEF2D antibody co-precipitated the DNA fragment containing the predicted MRE in the regulatory regions of the ACSL3 gene, indicating that MEF2D bound directly to the MRE in Huh7 cells (Figure 4F). We found that ACSL3 was highly expressed in HCC tissues (Figure 1H). Further analysis of RNA sequencing data from the TCGA database showed that MEF2D expression was also significantly elevated in HCC tissues compared to adjacent normal tissues (Figure 4G). To investigate the potential relationship between ACSL3 and MEF2D, we conducted a correlation analysis, which revealed a significant positive association between their expression levels in HCC (Figure 4H).

These results provide strong evidence for the ability of MEF2D to regulate the transcription of the ACSL3 gene.

## 4 Discussion

HCC is characterized by high morbidity and mortality rates, frequently developing in individuals with chronic hepatitis associated with viral infection, alcohol-induced liver damage, or metabolic syndrome. Despite significant advancements in the prevention, diagnosis, and treatment of HCC in recent years, overall survival rates remain low. Sorafenib is a first-line therapeutic agent for HCC; however, the rapid emergence of acquired resistance results in a significantly poor prognosis for HCC patients. Concurrently, NAFLD has emerged as a leading cause of cirrhosis and HCC (Byrne and Targher, 2015). Increasing research efforts have focused on the pathophysiology of NAFLD metabolic disorders and their progression to HCC (Foerster et al., 2022).

Ferroptosis is a novel type of iron-dependent cell death. Unlike other subtypes of programmed cell death, such as autophagy and apoptosis, ferroptosis is characterized by high levels of lipid peroxidation (Hassannia et al., 2019; Chen X. et al., 2021). The combined effects of excessive iron accumulation, oxidative stress and oxidative tissue injury are major factors that contribute to hepatocellular damage and disease progression (Pizzino et al., 2017). Ferroptosis occurs at multiple stages of liver disease, including hepatitis, liver fibrosis, cirrhosis, and primary liver cancer. In each condition, the induction of ferroptosis is associated with iron metabolism defects, imbalances in amino acid antioxidants, and the accumulation of lipid peroxides (Chen S. et al., 2021; Wu et al., 2021; Qi et al., 2020). Other studies have shown that ferroptosis and lipid metabolism disorders play a critical role in NASH, and that inhibiting ferroptosis can reduce the severity of NASH (Tsurusaki et al., 2019; Zhang et al., 2021).

Additionally, ferroptosis contributes to tumor drug resistance, particularly through the regulation of iron and lipid metabolism. For example, erastin resistance in acute myeloid leukemia (AML) cells can be reversed by inducing ferroptosis (Lu et al., 2017). In cisplatin-resistant gastric cancer, elevated ATF3 expression cooperates with erastin to inhibit the Keap1-Nrf2-xCT pathway, thereby promoting ferroptosis in cancer cells and reversing cisplatin resistance (Fu et al., 2021).

ACSL3, which converts exogenous MUFA into fatty acyl-CoAs, is essential for MUFA activation and promotes a ferroptosis-resistant cellular state (Magtanong et al., 2019; Ubellacker et al., 2020). More importantly, increasing evidence suggests that ACSL3 upregulation is associated with various diseases, including cancer. Our findings confirmed that ACSL3 expression was elevated in both NAFLD and HCC, and this aberrant expression contributed to the progression from NAFLD to HCC. Notably, we discovered that ACSL3 played a crucial role in regulating ferroptosis in HCC, with its high expression protecting HCC cells from ferroptosis damage. ACSL4 is recognized as a positive regulator of ferroptosis, with its high expression or increased activity enhancing cellular sensitivity to ferroptosis. During the process of ferroptosis in HCC, the interaction between ACSL3 and ACSL4 may be indirectly mediated through metabolic pathways or signaling pathways. Tumor cells might dynamically regulate the expression of ACSL3 and ACSL4 to fulfill specific metabolic demands or adapt to environmental stressors. This potential indirect metabolic complementation mechanism could manifest as a negative correlation between ACSL3 upregulation and ACSL4 downregulation. Interestingly, ACSL3 expression was also elevated in sorafenib-resistant HCC cells, and its increased expression was correlated with poor prognosis following sorafenib treatment. Compared to wild-type cells, HCC cells overexpressing ACSL3 exhibited higher cell viability and lower levels of ferroptosis after sorafenib treatment. The results of ACSL3 silencing in HCC cells were opposite to those described above.

To determine the direct regulatory mechanism of ACSL3 expression, we used bioinformatics analysis to identify MEF2D recognition elements in the regions upstream of the ACSL3 transcriptional sites. MEF2D, a master regulator of transcriptional reprogramming in cancer-related genes, has been reported to be overexpressed in HCC and is associated with tumor cell proliferation, invasion, and migration. By targeting MEF2D, our study demonstrated that MEF2D can bind to the promoter of ACSL3 and promote its expression. This study provides preliminary evidence supporting the regulatory role of MEF2D in HCC and highlights its potential molecular mechanisms. To accurately identify the binding sites of MEF2D and elucidate its regulatory relationship with ACSL3, promoter mutation experiments are required, along with an integrated analysis of clinical samples and animal models. These findings establish a solid foundation for further studies on the role of MEF2D in lipid metabolism dysregulation and tumor progression.

In conclusion, the present study confirmed the critical role of ACSL3 in sorafenib-induced ferroptosis. This finding highlights the great potential of targeting ACSL3 for the treatment of HCC. Prospective, multicenter clinical trials are still needed to validate these findings.

## Data Availability

The original contributions presented in the study are included in the article/Supplementary Material, further inquiries can be directed to the corresponding authors.
