# Supplementary material for: Transcription factor MEF2D regulates aberrant expression of ACSL3 and enhances sorafenib resistance by inhibiting ferroptosis in HCC
Source: Front Pharmacol. 2024 Dec 18;15:1464852. doi: 10.3389/fphar.2024.1464852 (PMC11688200; doi:10.3389/fphar.2024.1464852)
Supplement: Supplementary file 1 [file Table1.DOCX]

| TableS1. NAFLD patients information | | | | | | | | |
| --- | --- | --- | --- | --- | --- | --- | --- | --- |
| Age | Sex | Tumor  diameter(cm) | CNLC | BMI | NAFLD  Grade | AFP(ng/ml) | ALT(U/L） | AST（U/L） |
| 63 | female | 6.0*5.4 | CNLC Ⅰb | 28 | Middle | 1.64 | 11 | 14 |
| 40 | male | 10.0*9.1 | CNLC Ⅰb | 29 | High | 21 | 90 | 61 |
| 86 | male | 5.0*3.0 | CNLC Ⅰa | 24 | Low | 656 | 33 | 32 |

| TableS2. Positive correlation genes with ACSL3 | | | | | | |
| --- | --- | --- | --- | --- | --- | --- |
| Target  Gene/Attribute | Gene name | Spearman  Correlation | P-value | FDR  (BH) | Event_SD | Event_TD |
| [ACSL3](https://www.ncbi.nlm.nih.gov/gene/?term=ACSL3%5BSymbol%5D%20AND%20Homo%20sapiens) | acyl-CoA synthetase long chain family member 3 | 1.00E+00 | 1.00E-29 | 1.00E-25 | 3.71E+02 | 3.71E+02 |
| [SCD](https://www.ncbi.nlm.nih.gov/gene/?term=SCD%5BSymbol%5D%20AND%20Homo%20sapiens) | stearoyl-CoA desaturase | 5.41E-01 | 1.51E-29 | 1.50E-25 | 3.71E+02 | 3.71E+02 |
| [SREBF1](https://www.ncbi.nlm.nih.gov/gene/?term=SREBF1%5BSymbol%5D%20AND%20Homo%20sapiens) | sterol regulatory element binding transcription factor 1 | 5.36E-01 | 5.98E-29 | 3.97E-25 | 3.71E+02 | 3.71E+02 |
| [MID1IP1](https://www.ncbi.nlm.nih.gov/gene/?term=MID1IP1%5BSymbol%5D%20AND%20Homo%20sapiens) | MID1 interacting protein 1 | 5.03E-01 | 3.66E-25 | 1.83E-21 | 3.71E+02 | 3.71E+02 |
| [CCL20](https://www.ncbi.nlm.nih.gov/gene/?term=CCL20%5BSymbol%5D%20AND%20Homo%20sapiens) | C-C motif chemokine ligand 20 | 4.79E-01 | 1.16E-22 | 4.61E-19 | 3.71E+02 | 3.67E+02 |
| [AGPS](https://www.ncbi.nlm.nih.gov/gene/?term=AGPS%5BSymbol%5D%20AND%20Homo%20sapiens) | alkylglycerone phosphate synthase | 4.74E-01 | 3.91E-22 | 1.30E-18 | 3.71E+02 | 3.71E+02 |
| [PON2](https://www.ncbi.nlm.nih.gov/gene/?term=PON2%5BSymbol%5D%20AND%20Homo%20sapiens) | paraoxonase 2 | 4.68E-01 | 1.30E-21 | 3.69E-18 | 3.71E+02 | 3.71E+02 |
| [ACSL4](https://www.ncbi.nlm.nih.gov/gene/?term=ACSL4%5BSymbol%5D%20AND%20Homo%20sapiens) | acyl-CoA synthetase long chain family member 4 | 4.61E-01 | 6.20E-21 | 1.55E-17 | 3.71E+02 | 3.71E+02 |
| [CPD](https://www.ncbi.nlm.nih.gov/gene/?term=CPD%5BSymbol%5D%20AND%20Homo%20sapiens) | carboxypeptidase D | 4.59E-01 | 9.29E-21 | 2.06E-17 | 3.71E+02 | 3.71E+02 |
| [ROBO1](https://www.ncbi.nlm.nih.gov/gene/?term=ROBO1%5BSymbol%5D%20AND%20Homo%20sapiens) | roundabout guidance receptor 1 | 4.58E-01 | 1.22E-20 | 2.44E-17 | 3.71E+02 | 3.71E+02 |
| [NT5E](https://www.ncbi.nlm.nih.gov/gene/?term=NT5E%5BSymbol%5D%20AND%20Homo%20sapiens) | 5'-nucleotidase ecto | 4.48E-01 | 1.15E-19 | 2.09E-16 | 3.71E+02 | 3.71E+02 |
| [FNDC3B](https://www.ncbi.nlm.nih.gov/gene/?term=FNDC3B%5BSymbol%5D%20AND%20Homo%20sapiens) | fibronectin type III domain containing 3B | 4.41E-01 | 4.12E-19 | 6.85E-16 | 3.71E+02 | 3.71E+02 |
| [ACACA](https://www.ncbi.nlm.nih.gov/gene/?term=ACACA%5BSymbol%5D%20AND%20Homo%20sapiens) | acetyl-CoA carboxylase alpha | 4.20E-01 | 2.84E-17 | 4.35E-14 | 3.71E+02 | 3.71E+02 |
| [ETV5](https://www.ncbi.nlm.nih.gov/gene/?term=ETV5%5BSymbol%5D%20AND%20Homo%20sapiens) | ETS variant transcription factor 5 | 4.19E-01 | 3.48E-17 | 4.96E-14 | 3.71E+02 | 3.71E+02 |
| [CHD1L](https://www.ncbi.nlm.nih.gov/gene/?term=CHD1L%5BSymbol%5D%20AND%20Homo%20sapiens) | chromodomain helicase DNA binding protein 1 like | 4.06E-01 | 4.05E-16 | 5.04E-13 | 3.71E+02 | 3.71E+02 |
| [SERPINB1](https://www.ncbi.nlm.nih.gov/gene/?term=SERPINB1%5BSymbol%5D%20AND%20Homo%20sapiens) | serpin family B member 1 | 4.05E-01 | 4.79E-16 | 5.61E-13 | 3.71E+02 | 3.71E+02 |
| [PLOD2](https://www.ncbi.nlm.nih.gov/gene/?term=PLOD2%5BSymbol%5D%20AND%20Homo%20sapiens) | procollagen-lysine,2-oxoglutarate 5-dioxygenase 2 | 4.03E-01 | 6.59E-16 | 7.30E-13 | 3.71E+02 | 3.71E+02 |
| [MGC12916](https://www.ncbi.nlm.nih.gov/gene/?term=MGC12916%5BSymbol%5D%20AND%20Homo%20sapiens) | uncharacterized protein MGC12916 | 3.92E-01 | 4.76E-15 | 5.00E-12 | 3.71E+02 | 3.70E+02 |
| [AGTR1](https://www.ncbi.nlm.nih.gov/gene/?term=AGTR1%5BSymbol%5D%20AND%20Homo%20sapiens) | Type-1 angiotensin II receptor | 3.85E-01 | 1.41E-14 | 1.27E-11 | 3.71E+02 | 3.71E+02 |
| [ABCA1](https://www.ncbi.nlm.nih.gov/gene/?term=ABCA1%5BSymbol%5D%20AND%20Homo%20sapiens) | ATP binding cassette subfamily A member 1 | 3.83E-01 | 2.07E-14 | 1.65E-11 | 3.71E+02 | 3.71E+02 |
| [TMC7](https://www.ncbi.nlm.nih.gov/gene/?term=TMC7%5BSymbol%5D%20AND%20Homo%20sapiens) | transmembrane channel like 7 | 3.81E-01 | 2.74E-14 | 2.10E-11 | 3.71E+02 | 3.71E+02 |
| [DUSP6](https://www.ncbi.nlm.nih.gov/gene/?term=DUSP6%5BSymbol%5D%20AND%20Homo%20sapiens) | dual specificity phosphatase 6 | 3.80E-01 | 3.47E-14 | 2.56E-11 | 3.71E+02 | 3.71E+02 |
| [EPS8L3](https://www.ncbi.nlm.nih.gov/gene/?term=EPS8L3%5BSymbol%5D%20AND%20Homo%20sapiens) | epidermal growth factor receptor kinase substrate 8-like  protein 3 | 3.80E-01 | 3.71E-14 | 2.64E-11 | 3.71E+02 | 3.52E+02 |
| [SREBF2](https://www.ncbi.nlm.nih.gov/gene/?term=SREBF2%5BSymbol%5D%20AND%20Homo%20sapiens) | sterol regulatory element binding transcription factor 2 | 3.78E-01 | 4.74E-14 | 3.26E-11 | 3.71E+02 | 3.71E+02 |
| [AZIN1](https://www.ncbi.nlm.nih.gov/gene/?term=AZIN1%5BSymbol%5D%20AND%20Homo%20sapiens) | antizyme inhibitor 1 | 3.76E-01 | 6.31E-14 | 4.19E-11 | 3.71E+02 | 3.71E+02 |
| [SLC4A7](https://www.ncbi.nlm.nih.gov/gene/?term=SLC4A7%5BSymbol%5D%20AND%20Homo%20sapiens) | solute carrier family 4 member 7 | 3.76E-01 | 7.17E-14 | 4.44E-11 | 3.71E+02 | 3.71E+02 |
| [ADM2](https://www.ncbi.nlm.nih.gov/gene/?term=ADM2%5BSymbol%5D%20AND%20Homo%20sapiens) | adrenomedullin 2 | 3.75E-01 | 7.35E-14 | 4.44E-11 | 3.71E+02 | 3.70E+02 |
| [MMD](https://www.ncbi.nlm.nih.gov/gene/?term=MMD%5BSymbol%5D%20AND%20Homo%20sapiens) | [monocyte to macrophage differentiation associated](https://www.ncbi.nlm.nih.gov/gene/?term=MMD%5BSymbol%5D%20AND%20Homo%20sapiens) | 3.75E-01 | 7.42E-14 | 4.44E-11 | 3.71E+02 | 3.71E+02 |
| [S100P](https://www.ncbi.nlm.nih.gov/gene/?term=S100P%5BSymbol%5D%20AND%20Homo%20sapiens) | S100 calcium binding protein P | 3.75E-01 | 8.39E-14 | 4.64E-11 | 3.71E+02 | 3.59E+02 |
| [ATAD2](https://www.ncbi.nlm.nih.gov/gene/?term=ATAD2%5BSymbol%5D%20AND%20Homo%20sapiens) | ATPase family AAA domain containing 2 | 3.74E-01 | 8.69E-14 | 4.64E-11 | 3.71E+02 | 3.71E+02 |
| [ETV4](https://www.ncbi.nlm.nih.gov/gene/?term=ETV4%5BSymbol%5D%20AND%20Homo%20sapiens) | ETS variant transcription factor 4 | 3.74E-01 | 8.60E-14 | 4.64E-11 | 3.71E+02 | 3.68E+02 |
| [LYZ](https://www.ncbi.nlm.nih.gov/gene/?term=LYZ%5BSymbol%5D%20AND%20Homo%20sapiens) | lysozyme | 3.74E-01 | 8.86E-14 | 4.64E-11 | 3.71E+02 | 3.71E+02 |
| [ACVR1](https://www.ncbi.nlm.nih.gov/gene/?term=ACVR1%5BSymbol%5D%20AND%20Homo%20sapiens) | activin A receptor type 1 | 3.69E-01 | 1.99E-13 | 9.43E-11 | 3.71E+02 | 3.71E+02 |
| [ZDHHC9](https://www.ncbi.nlm.nih.gov/gene/?term=ZDHHC9%5BSymbol%5D%20AND%20Homo%20sapiens) | zinc finger DHHC-type palmitoyltransferase 9 | 3.69E-01 | 2.24E-13 | 1.01E-10 | 3.71E+02 | 3.71E+02 |
| [TDGF3](https://www.ncbi.nlm.nih.gov/gene/?term=TDGF3%5BSymbol%5D%20AND%20Homo%20sapiens) | teratocarcinoma-derived growth factor 1 pseudogene 3 | 3.68E-01 | 2.27E-13 | 1.01E-10 | 3.71E+02 | 3.16E+02 |
| [PAG1](https://www.ncbi.nlm.nih.gov/gene/?term=PAG1%5BSymbol%5D%20AND%20Homo%20sapiens) | phosphoprotein membrane anchor with  glycosphingolipid microdomains 1 | 3.68E-01 | 2.45E-13 | 1.06E-10 | 3.71E+02 | 3.71E+02 |
| [CCR6](https://www.ncbi.nlm.nih.gov/gene/?term=CCR6%5BSymbol%5D%20AND%20Homo%20sapiens) | C-C motif chemokine receptor 6 | 3.68E-01 | 2.49E-13 | 1.06E-10 | 3.71E+02 | 3.70E+02 |
| [GORASP2](https://www.ncbi.nlm.nih.gov/gene/?term=GORASP2%5BSymbol%5D%20AND%20Homo%20sapiens) | golgi reassembly stacking protein 2 | 3.68E-01 | 2.55E-13 | 1.06E-10 | 3.71E+02 | 3.71E+02 |
| [STEAP1](https://www.ncbi.nlm.nih.gov/gene/?term=STEAP1%5BSymbol%5D%20AND%20Homo%20sapiens) | STEAP family member 1 | 3.67E-01 | 2.92E-13 | 1.19E-10 | 3.71E+02 | 3.69E+02 |
| [KIF21B](https://www.ncbi.nlm.nih.gov/gene/?term=KIF21B%5BSymbol%5D%20AND%20Homo%20sapiens) | kinesin family member 21B | 3.66E-01 | 3.23E-13 | 1.29E-10 | 3.71E+02 | 3.71E+02 |
| [FAM60A](https://www.ncbi.nlm.nih.gov/gene/?term=FAM60A%5BSymbol%5D%20AND%20Homo%20sapiens) | [family with sequence similarity 60 member A](https://www.ncbi.nlm.nih.gov/gene/418140) | 3.66E-01 | 3.64E-13 | 1.38E-10 | 3.71E+02 | 3.71E+02 |
| [SHC1](https://www.ncbi.nlm.nih.gov/gene/?term=SHC1%5BSymbol%5D%20AND%20Homo%20sapiens) | SHC adaptor protein 1 | 3.65E-01 | 3.69E-13 | 1.38E-10 | 3.71E+02 | 3.71E+02 |
| [TDGF1](https://www.ncbi.nlm.nih.gov/gene/?term=TDGF1%5BSymbol%5D%20AND%20Homo%20sapiens) | teratocarcinoma-derived growth factor 1 | 3.65E-01 | 3.68E-13 | 1.38E-10 | 3.71E+02 | 3.61E+02 |
| [FLVCR1](https://www.ncbi.nlm.nih.gov/gene/?term=FLVCR1%5BSymbol%5D%20AND%20Homo%20sapiens) | FLVCR heme transporter 1 | 3.65E-01 | 3.73E-13 | 1.38E-10 | 3.71E+02 | 3.71E+02 |
| [EPRS](https://www.ncbi.nlm.nih.gov/gene/?term=EPRS%5BSymbol%5D%20AND%20Homo%20sapiens) | glutamyl-prolyl-tRNA synthetase | 3.65E-01 | 4.09E-13 | 1.48E-10 | 3.71E+02 | 3.71E+02 |
| [HSPD1](https://www.ncbi.nlm.nih.gov/gene/?term=HSPD1%5BSymbol%5D%20AND%20Homo%20sapiens) | heat shock protein family D (Hsp60) member 1 | 3.64E-01 | 4.71E-13 | 1.68E-10 | 3.71E+02 | 3.71E+02 |
| [MPZL1](https://www.ncbi.nlm.nih.gov/gene/?term=MPZL1%5BSymbol%5D%20AND%20Homo%20sapiens) | myelin protein zero like 1 | 3.64E-01 | 4.91E-13 | 1.72E-10 | 3.71E+02 | 3.71E+02 |
| [TRNP1](https://www.ncbi.nlm.nih.gov/gene/?term=TRNP1%5BSymbol%5D%20AND%20Homo%20sapiens) | TMF1-regulated nuclear protein 1 | 3.63E-01 | 5.71E-13 | 1.89E-10 | 3.71E+02 | 3.71E+02 |
| [SPRED2](https://www.ncbi.nlm.nih.gov/gene/?term=SPRED2%5BSymbol%5D%20AND%20Homo%20sapiens) | sprouty-related EVH1 domain containing 2 | 3.61E-01 | 7.65E-13 | 2.42E-10 | 3.71E+02 | 3.71E+02 |
| [MPP7](https://www.ncbi.nlm.nih.gov/gene/?term=MPP7%5BSymbol%5D%20AND%20Homo%20sapiens) | MAGUK p55 scaffold protein 7 | 3.61E-01 | 7.82E-13 | 2.44E-10 | 3.71E+02 | 3.71E+02 |

| TableS3: Negative correlation genes with ACSL3 | | | | | | |
| --- | --- | --- | --- | --- | --- | --- |
| Target  Gene/Attribute | Name | Spearman  Correlation | P-value | FDR (BH) | Event_SD | Event_TD |
| [FNDC5](https://www.ncbi.nlm.nih.gov/gene/?term=FNDC5%5BSymbol%5D%20AND%20Homo%20sapiens) | [fibronectin type III domain containing 5](https://www.ncbi.nlm.nih.gov/gene/252995) | -4.15E-01 | 7.54E-17 | 1.00E-13 | 3.71E+02 | 3.71E+02 |
| [APBA1](https://www.ncbi.nlm.nih.gov/gene/?term=APBA1%5BSymbol%5D%20AND%20Homo%20sapiens) | amyloid beta precursor protein binding family A member 1 | -3.90E-01 | 6.80E-15 | 6.78E-12 | 3.71E+02 | 3.71E+02 |
| [SLC25A34](https://www.ncbi.nlm.nih.gov/gene/?term=SLC25A34%5BSymbol%5D%20AND%20Homo%20sapiens) | solute carrier family 25 member 34 | -3.87E-01 | 9.97E-15 | 9.46E-12 | 3.71E+02 | 3.71E+02 |
| [ARMC5](https://www.ncbi.nlm.nih.gov/gene/?term=ARMC5%5BSymbol%5D%20AND%20Homo%20sapiens) | armadillo repeat containing 5 | -3.85E-01 | 1.47E-14 | 1.28E-11 | 3.71E+02 | 3.71E+02 |
| [PKIG](https://www.ncbi.nlm.nih.gov/gene/?term=PKIG%5BSymbol%5D%20AND%20Homo%20sapiens) | cAMP-dependent protein kinase inhibitor gamma | -3.85E-01 | 1.57E-14 | 1.30E-11 | 3.71E+02 | 3.71E+02 |
| [MAPK8IP1](https://www.ncbi.nlm.nih.gov/gene/?term=MAPK8IP1%5BSymbol%5D%20AND%20Homo%20sapiens) | mitogen-activated protein kinase 8 interacting protein 1 | -3.75E-01 | 7.57E-14 | 4.44E-11 | 3.71E+02 | 3.71E+02 |
| [PRODH](https://www.ncbi.nlm.nih.gov/gene/?term=PRODH%5BSymbol%5D%20AND%20Homo%20sapiens) | proline dehydrogenase 1 | -3.73E-01 | 1.09E-13 | 5.57E-11 | 3.71E+02 | 3.69E+02 |
| [MAD1L1](https://www.ncbi.nlm.nih.gov/gene/?term=MAD1L1%5BSymbol%5D%20AND%20Homo%20sapiens) | mitotic arrest deficient 1 like 1 | -3.72E-01 | 1.22E-13 | 6.10E-11 | 3.71E+02 | 3.71E+02 |
| [ACBD4](https://www.ncbi.nlm.nih.gov/gene/?term=ACBD4%5BSymbol%5D%20AND%20Homo%20sapiens) | acyl-CoA binding domain containing 4 | -3.71E-01 | 1.54E-13 | 7.46E-11 | 3.71E+02 | 3.71E+02 |
| [SPSB3](https://www.ncbi.nlm.nih.gov/gene/?term=SPSB3%5BSymbol%5D%20AND%20Homo%20sapiens) | splA/ryanodine receptor domain and SOCS box containing 3 | -3.68E-01 | 2.27E-13 | 1.01E-10 | 3.71E+02 | 3.71E+02 |
| [C11orf66](https://www.ncbi.nlm.nih.gov/gene/?term=C11orf66%5BSymbol%5D%20AND%20Homo%20sapiens) | protein phosphatase 1 regulatory subunit 32 | -3.63E-01 | 5.15E-13 | 1.77E-10 | 3.71E+02 | 3.71E+02 |
| [FAM54B](https://www.ncbi.nlm.nih.gov/gene/?term=FAM54B%5BSymbol%5D%20AND%20Homo%20sapiens) | mitochondrial fission regulator 1 like | -3.63E-01 | 5.30E-13 | 1.79E-10 | 3.71E+02 | 3.71E+02 |
| [MFSD2A](https://www.ncbi.nlm.nih.gov/gene/?term=MFSD2A%5BSymbol%5D%20AND%20Homo%20sapiens) | major facilitator superfamily domain containing 2A | -3.62E-01 | 6.77E-13 | 2.21E-10 | 3.71E+02 | 3.68E+02 |
| [C21orf119](https://www.ncbi.nlm.nih.gov/gene/?term=C21orf119%5BSymbol%5D%20AND%20Homo%20sapiens) | URB1 antisense RNA 1 | -3.61E-01 | 7.00E-13 | 2.25E-10 | 3.71E+02 | 3.71E+02 |
| [FAAH](https://www.ncbi.nlm.nih.gov/gene/?term=FAAH%5BSymbol%5D%20AND%20Homo%20sapiens) | fatty acid amide hydrolase | -3.61E-01 | 7.96E-13 | 2.44E-10 | 3.71E+02 | 3.71E+02 |
| [SLC10A1](https://www.ncbi.nlm.nih.gov/gene/?term=SLC10A1%5BSymbol%5D%20AND%20Homo%20sapiens) | solute carrier family 10 member 1 | -3.55E-01 | 1.81E-12 | 4.94E-10 | 3.71E+02 | 3.68E+02 |
| [AMY2B](https://www.ncbi.nlm.nih.gov/gene/?term=AMY2B%5BSymbol%5D%20AND%20Homo%20sapiens) | amylase alpha 2B | -3.53E-01 | 2.64E-12 | 7.00E-10 | 3.71E+02 | 3.71E+02 |
| [CLDN14](https://www.ncbi.nlm.nih.gov/gene/?term=CLDN14%5BSymbol%5D%20AND%20Homo%20sapiens) | claudin 14 | -3.51E-01 | 3.29E-12 | 8.50E-10 | 3.71E+02 | 3.70E+02 |
| [GTF2IRD2](https://www.ncbi.nlm.nih.gov/gene/?term=GTF2IRD2%5BSymbol%5D%20AND%20Homo%20sapiens) | GTF2I repeat domain containing 2 | -3.50E-01 | 3.92E-12 | 9.92E-10 | 3.71E+02 | 3.71E+02 |
| [PEX11G](https://www.ncbi.nlm.nih.gov/gene/?term=PEX11G%5BSymbol%5D%20AND%20Homo%20sapiens) | peroxisomal biogenesis factor 11 gamma | -3.50E-01 | 3.94E-12 | 9.92E-10 | 3.71E+02 | 3.71E+02 |
| [MACROD1](https://www.ncbi.nlm.nih.gov/gene/?term=MACROD1%5BSymbol%5D%20AND%20Homo%20sapiens) | mono-ADP ribosylhydrolase 1 | -3.48E-01 | 5.35E-12 | 1.32E-09 | 3.71E+02 | 3.71E+02 |
| [AMY1A](https://www.ncbi.nlm.nih.gov/gene/?term=AMY1A%5BSymbol%5D%20AND%20Homo%20sapiens) | amylase alpha 1A | -3.46E-01 | 7.08E-12 | 1.70E-09 | 3.71E+02 | 3.49E+02 |
| [GPR137](https://www.ncbi.nlm.nih.gov/gene/?term=GPR137%5BSymbol%5D%20AND%20Homo%20sapiens) | G protein-coupled receptor 137 | -3.46E-01 | 7.69E-12 | 1.80E-09 | 3.71E+02 | 3.71E+02 |
| [GSTM4](https://www.ncbi.nlm.nih.gov/gene/?term=GSTM4%5BSymbol%5D%20AND%20Homo%20sapiens) | glutathione S-transferase mu 4 | -3.44E-01 | 9.59E-12 | 2.15E-09 | 3.71E+02 | 3.71E+02 |
| [CYP7A1](https://www.ncbi.nlm.nih.gov/gene/?term=CYP7A1%5BSymbol%5D%20AND%20Homo%20sapiens) | cytochrome P450 family 7 subfamily A member 1 | -3.43E-01 | 1.08E-11 | 2.36E-09 | 3.71E+02 | 3.62E+02 |
| [ECHDC2](https://www.ncbi.nlm.nih.gov/gene/?term=ECHDC2%5BSymbol%5D%20AND%20Homo%20sapiens) | enoyl-CoA hydratase domain containing 2 | -3.43E-01 | 1.11E-11 | 2.41E-09 | 3.71E+02 | 3.71E+02 |
| [LOC388387](https://www.ncbi.nlm.nih.gov/gene/?term=LOC388387%5BSymbol%5D%20AND%20Homo%20sapiens) | long intergenic non-protein coding RNA 671 | -3.42E-01 | 1.30E-11 | 2.76E-09 | 3.71E+02 | 3.65E+02 |
| [ZNF688](https://www.ncbi.nlm.nih.gov/gene/?term=ZNF688%5BSymbol%5D%20AND%20Homo%20sapiens) | zinc finger protein 688 | -3.41E-01 | 1.56E-11 | 3.18E-09 | 3.71E+02 | 3.71E+02 |
| [TCAP](https://www.ncbi.nlm.nih.gov/gene/?term=TCAP%5BSymbol%5D%20AND%20Homo%20sapiens) | titin-cap | -3.39E-01 | 2.08E-11 | 4.04E-09 | 3.71E+02 | 3.68E+02 |
| [FGGY](https://www.ncbi.nlm.nih.gov/gene/?term=FGGY%5BSymbol%5D%20AND%20Homo%20sapiens) | FGGY carbohydrate kinase domain containing | -3.37E-01 | 2.53E-11 | 4.63E-09 | 3.71E+02 | 3.71E+02 |
| [GATSL3](https://www.ncbi.nlm.nih.gov/gene/?term=GATSL3%5BSymbol%5D%20AND%20Homo%20sapiens) | cytosolic arginine sensor for mTORC1 subunit 1 | -3.37E-01 | 2.63E-11 | 4.77E-09 | 3.71E+02 | 3.71E+02 |
| [SORBS2](https://www.ncbi.nlm.nih.gov/gene/?term=SORBS2%5BSymbol%5D%20AND%20Homo%20sapiens) | sorbin and SH3 domain containing 2 | -3.37E-01 | 2.69E-11 | 4.82E-09 | 3.71E+02 | 3.71E+02 |
| [ID2](https://www.ncbi.nlm.nih.gov/gene/?term=ID2%5BSymbol%5D%20AND%20Homo%20sapiens) | inhibitor of DNA binding 2 | -3.37E-01 | 2.83E-11 | 5.04E-09 | 3.71E+02 | 3.71E+02 |
| [DHRS4L1](https://www.ncbi.nlm.nih.gov/gene/?term=DHRS4L1%5BSymbol%5D%20AND%20Homo%20sapiens) | dehydrogenase/reductase 4 like 1 (pseudogene) | -3.36E-01 | 3.15E-11 | 5.42E-09 | 3.71E+02 | 3.71E+02 |
| [ZNF833](https://www.ncbi.nlm.nih.gov/gene/?term=ZNF833%5BSymbol%5D%20AND%20Homo%20sapiens) | zinc finger protein 833, pseudogene | -3.36E-01 | 3.16E-11 | 5.42E-09 | 3.71E+02 | 3.70E+02 |
| [PCSK4](https://www.ncbi.nlm.nih.gov/gene/?term=PCSK4%5BSymbol%5D%20AND%20Homo%20sapiens) | proprotein convertase subtilisin/kexin type 4 | -3.34E-01 | 3.81E-11 | 6.22E-09 | 3.71E+02 | 3.71E+02 |
| [HAGH](https://www.ncbi.nlm.nih.gov/gene/?term=HAGH%5BSymbol%5D%20AND%20Homo%20sapiens) | hydroxyacylglutathione hydrolase | -3.34E-01 | 4.32E-11 | 6.88E-09 | 3.71E+02 | 3.71E+02 |
| [TCEA2](https://www.ncbi.nlm.nih.gov/gene/?term=TCEA2%5BSymbol%5D%20AND%20Homo%20sapiens) | transcription elongation factor A2 | -3.33E-01 | 4.66E-11 | 7.31E-09 | 3.71E+02 | 3.71E+02 |
| [NT5M](https://www.ncbi.nlm.nih.gov/gene/?term=NT5M%5BSymbol%5D%20AND%20Homo%20sapiens) | 5',3'-nucleotidase, mitochondrial | -3.32E-01 | 5.28E-11 | 8.02E-09 | 3.71E+02 | 3.71E+02 |
| [ETNK2](https://www.ncbi.nlm.nih.gov/gene/?term=ETNK2%5BSymbol%5D%20AND%20Homo%20sapiens) | ethanolamine kinase 2 | -3.32E-01 | 5.41E-11 | 8.16E-09 | 3.71E+02 | 3.71E+02 |
| [ZNF771](https://www.ncbi.nlm.nih.gov/gene/?term=ZNF771%5BSymbol%5D%20AND%20Homo%20sapiens) | zinc finger protein 771 | -3.29E-01 | 7.81E-11 | 1.11E-08 | 3.71E+02 | 3.71E+02 |

| [FITM1](https://www.ncbi.nlm.nih.gov/gene/?term=FITM1%5BSymbol%5D%20AND%20Homo%20sapiens) | fat storage inducing transmembrane protein 1 | -3.29E-01 | 8.38E-11 | 1.18E-08 | 3.71E+02 | 3.71E+02 |
| --- | --- | --- | --- | --- | --- | --- |
| [CSRP1](https://www.ncbi.nlm.nih.gov/gene/?term=CSRP1%5BSymbol%5D%20AND%20Homo%20sapiens) | cysteine and glycine rich protein 1 | -3.27E-01 | 1.13E-10 | 1.54E-08 | 3.71E+02 | 3.71E+02 |
| [KCNE2](https://www.ncbi.nlm.nih.gov/gene/?term=KCNE2%5BSymbol%5D%20AND%20Homo%20sapiens) | potassium voltage-gated channel subfamily E regulatory  subunit 2 | -3.27E-01 | 1.16E-10 | 1.56E-08 | 3.71E+02 | 3.69E+02 |
| [PXMP2](https://www.ncbi.nlm.nih.gov/gene/?term=PXMP2%5BSymbol%5D%20AND%20Homo%20sapiens) | peroxisomal membrane protein 2 | -3.26E-01 | 1.19E-10 | 1.59E-08 | 3.71E+02 | 3.71E+02 |
| [CYP8B1](https://www.ncbi.nlm.nih.gov/gene/?term=CYP8B1%5BSymbol%5D%20AND%20Homo%20sapiens) | cytochrome P450 family 8 subfamily B member 1 | -3.26E-01 | 1.25E-10 | 1.65E-08 | 3.71E+02 | 3.71E+02 |
| [PRKAG2](https://www.ncbi.nlm.nih.gov/gene/?term=PRKAG2%5BSymbol%5D%20AND%20Homo%20sapiens) | protein kinase AMP-activated non-catalytic subunit gamma  2 | -3.25E-01 | 1.39E-10 | 1.79E-08 | 3.71E+02 | 3.71E+02 |
| [ZNF653](https://www.ncbi.nlm.nih.gov/gene/?term=ZNF653%5BSymbol%5D%20AND%20Homo%20sapiens) | zinc finger protein 653 | -3.25E-01 | 1.41E-10 | 1.81E-08 | 3.71E+02 | 3.71E+02 |
| [HAAO](https://www.ncbi.nlm.nih.gov/gene/?term=HAAO%5BSymbol%5D%20AND%20Homo%20sapiens) | 3-hydroxyanthranilate 3,4-dioxygenase | -3.25E-01 | 1.42E-10 | 1.81E-08 | 3.71E+02 | 3.71E+02 |
| [PNMT](https://www.ncbi.nlm.nih.gov/gene/?term=PNMT%5BSymbol%5D%20AND%20Homo%20sapiens) | phosphatidylethanolamine N-methyltransferase | -3.24E-01 | 1.66E-10 | 2.08E-08 | 3.71E+02 | 2.43E+02 |
